# Identification and expression analysis of cinnamyl CoA reductase gene family and function of *StCCR6* in potato (*Solanum tuberosum* L.)

**DOI:** 10.3389/fpls.2025.1638073

**Published:** 2025-08-11

**Authors:** Chong Du, Yunzhu Che, Zengli Zhang, Fumeng He, Xinqi Zhang, Yujie Han, Zelin Yang, Jiaqi Wang, Tianshuai Qi, Ying Lan, Yingnan Wang, Fenglan Li

**Affiliations:** ^1^ College of Life Sciences, Northeast Agricultural University, Harbin, Heilongjiang, China; ^2^ Qiqihar Branch, Heilongjiang Academy of Agricultural Sciences, Qiqihar, Heilongjiang, China

**Keywords:** potato, gene family, CCR, abiotic stress, gene expression, VIGS

## Abstract

Cinnamoyl-CoA reductase (CCR), a key rate-limiting enzyme in plant lignin biosynthesis, critically regulates plant growth, development, and abiotic stress responses. As one of the world’s four major staple crops, potato (*Solanum tuberosum* L.) is extensively cultivated due to its nutritional value and versatile applications, underscoring the importance of investigating the *StCCR* gene family and its expression patterns under abiotic stress. In this study, we identified 10 *CCR* genes from the Atlantic potato genome and conducted comprehensive analyses of their phylogenetic relationships, gene structures, species collinearity, cis-regulatory elements, and expression specificity. Virus-induced gene silencing (VIGS) was used to silence *StCCR6*, resulting in altered lignin content and enhanced susceptibility to bacterial infection. Results revealed structural and functional divergences among *StCCRs* members. Tissue-specific expression profiling demonstrated higher transcript abundance in stems and leaves compared to roots. Notably, *StCCRs* exhibited differential expression patterns across multiple stress conditions, with Subfamily I genes showing consistent upregulation under various treatments, suggesting their potential as core candidates mediating stress-responsive lignification. Silencing of *StCCR6* altered lignin content and cell wall structure in potato, and the oxidative damage was more serious after bacterial infection. These findings establish a foundation for elucidating the functional roles of *StCCRs* in potato growth regulation and stress adaptation mechanisms.

## 1 Introduction

Potato (*Solanum tuberosum* L.), recognized as one of the world’s four major staple crops and a vital non-cereal food source (Staiger, 2008), faces significant challenges during cultivation due to susceptibility to abiotic stresses such as high temperature and drought (Tang et al., 2018; Nasir and Toth, 2022). These constraints frequently impair normal growth, leading to substantial yield reductions and compromising tuber commercial viability. Consequently, enhancing stress resilience in potato has become imperative to safeguard agricultural productivity and industrial sustainability.

Plants have evolved intrinsic survival mechanisms to combat biotic and abiotic stresses through dynamic structural modifications of cell walls, establishing passive defense barriers (Bacete et al., 2018). Lignin, as a key structural component of plant cell walls, fulfills critical physiological roles by reinforcing cellular integrity, reducing water permeability and transpiration rates, and maintaining osmotic equilibrium and membrane stability (Boerjan et al., 2003). The phenylpropanoid metabolic pathway generates three lignin monomers that polymerize into lignin polymers, which deposit within cell walls to form compact secondary cell walls. This lignification process enhances mechanical strength, confers structural defense characteristics, and facilitates resistance against pathogenic invasion and environmental stressors (Chappie, 2010; Dixon and Barros, 2019; Goujon et al., 2003; Dong and Lin, 2021; Lv, 2022). The lignin monomer biosynthesis pathway involves multiple key enzymes and genes associated with phenylpropanoid metabolism. *CCR*, the first committed enzyme in the lignin-specific synthesis pathway, acts as a gatekeeper regulating carbon flux into lignin biosynthesis and serves as a critical rate-limiting enzyme in lignification (Li et al., 2010). Genetic modifications of *CCR* genes typically induce global alterations in lignin content across all three branches of the lignin biosynthetic pathway, significantly impacting plant growth and development (Tamasloukht et al., 2011; Tu et al., 2010). Notably, *AtCCR1* knockout in *Arabidopsis thaliana* resulted in a 50% reduction in stem lignin content compared to wild-type plants, accompanied by developmental retardation (Jones et al., 2001). Similarly, transgenic tobacco (*Nicotiana benthamiana*) with severely downregulated *CCR* activity exhibited not only diminished lignin deposition but also phenotypic abnormalities including dwarfism, leaf malformation, and vascular collapse (Baucher et al., 2003). Conversely, upregulated *OsCCR* expression in rice enhances lignin monomer biosynthesis for cell wall fortification, thereby improving resistance to pathogen invasion (Kawasaki et al., 2006).

Members of the *CCR* gene family exhibit tissue-specific expression patterns across plant species. In wheat (*Triticum aestivum*), *TaCCR1* shows negligible expression in roots (Ma, 2007), whereas *TaCCR2* demonstrates high transcript abundance (Ma and Tian, 2005). A similar divergence occurs in maize (*Zea mays*), with *ZmCCR1* predominating in roots over minimally expressed *ZmCCR2* (Pichon et al., 1998), a pattern paralleled in *Arabidopsis thaliana* (Lauvergeat et al., 2001). Striking tissue specificity is observed in trembling aspen (*Populus tremuloides*), where *CCR* expression remains undetectable in leaves but reaches peak levels in mature stems (Li et al., 2005). The content of *CCR* in stems of eucalyptus (*Eucalyptus* spp.) gradually increased with the growth of stems, and the expression of *CCR* in old stems was higher than that in young stems, with little expression in leaves and almost no expression in seeds (Lacombe et al., 2010). These collective findings highlight the spatiotemporal regulatory specificity of *CCR* genes during plant development.

Lignification of plant cell walls, as demonstrated by Dixon and Lamb (1990), enhances mechanical strength and confers resistance to environmental stressors and pathogens. It was found that in *Toon sinensis* and celery (*Apium graveolens*) (Sui et al., 2018; Wu et al., 2016), *TsCCR* and *AgCCR* genes were induced by low temperature, high temperature, drought and salt stress. Similarly, *GmCCR* in soybean exhibits strong upregulation under drought and salt stress, with mild induction by localized and systemic wounding (So et al., 2010). Phytohormone-mediated stress mitigation involves enhanced antioxidant enzyme activity for free radical scavenging and osmoregulatory compound accumulation (Yang et al., 2022; Yan and Dong, 2014). Abscisic acid (ABA) activates CCR expression via signaling cascades to boost lignin biosynthesis under stress conditions (Liu et al., 2021). While ABA downregulates *IiCCR* expression during plant cultivation, Gibberellin (GA3) significantly upregulates its transcript levels, as evidenced by promoter activity assays of pepper *CcCCR2* under ABA/GA3 treatments (Wu, 2022; Hu et al., 2011). These findings collectively implicate GA3 as a potential signaling molecule orchestrating CCR-mediated stress adaptation mechanisms. Pathogen-responsive regulation of *CCR* genes has been documented across plant-pathogen interactions (Prasad et al., 2011). In switchgrass (*Panicum virgatum*) infected with rust pathogens, *PvCCR2* exhibited 3.5-fold induction in leaves at 10 days post-inoculation (dpi), while *PvCCR1* remained uninduced, suggesting subfunctionalization in biotic stress adaptation (Escamilla-Treviño et al., 2010). Contrasting regulatory patterns emerge in *Brassica oleracea*, *SmCCR-2* expression initially declines upon *Xanthomonas campestris* infection and then showed a periodic fluctuation trend with the increase of infection time (Chen et al., 2011). Comparative studies of powdery mildew-resistant squash cultivars revealed sustained *CCR* upregulation with prolonged activation kinetics in resistant genotypes post-infection (Chen, 2013). These collective findings underscore the multifunctional importance of *CCR* genes in coordinating developmental processes and abiotic/biotic stress tolerance mechanisms.

However, the completed genome sequencing of *Solanum tuberosum* L. ‘Atlantic’ has enabled comprehensive genomic analysis of the *CCR* gene family in potato. This study systematically identified potato *StCCR* genes and characterized their structural features, phylogenetic relationships, and evolutionary divergence. Tissue-specific expression profiling revealed differential *StCCR* transcript abundance in roots, stems, and leaves. Furthermore, by analyzing *StCCR* expression patterns under diverse abiotic stresses (high/low temperature, NaCl, mannitol) and phytohormone treatments (ABA, indole-3-acetic acid (IAA), GA3), we identified key *StCCR* candidates potentially involved in growth regulation and stress adaptation. In addition, we further cloned and expressed recombinant *StCCR6*, performed subcellular localization analysis and silenced it in potato to preliminatively explore its function. These findings provide critical insights into the functional specialization of *CCR* genes during potato development and establish a theoretical foundation for further mechanistic investigations.

## 2 Materials and methods

### 2.1 Identification, physicochemical properties and subcellular localization prediction analysis of CCR gene

The Atlantic potato genome, annotation file, protein sequence and other information were obtained from potato database (). We used PFam (https://pfam.xfam.org/) to download the HMM model of the conserved domain (PF01370) in CCR protein (Jaina et al., 2020) to identify the potato *CCR* gene family genome-wide. The potato protein sequence was compared with Arabidopsis CCR protein sequence in NCBI database by BLASTP. By online website (http://eggnog-mapper.embl.de/), eggNOG HMMER3.0 (http://hmmer.org/download.html) (Jaina et al., 2013), the NCBI - CDD (Aron et al., 2015) of preliminary screening genes annotated and search, etc. After comprehensive analysis, we finally identified 10 *StCCR* genes, and these potato *CCR* genes were renamed *StCCRs*. According to the gene location relationship on the chromosome, they were identified as *StCCR1* to *StCCR10*. For example, The gene ID Soltu.Atl.03_1G016980.1 has been named *StCCR1*. The amino acid number, molecular weight, isoelectric point and hydrophobic index of potato *StCCR* members were analyzed by ExPASy (https://www.expasy.org/) (Elisabeth et al., 2003). WoLF PSORT(https://wolfpsort.hgc.jp) was used to predict protein subcellular localization (Paul et al., 2007).

### 2.2 Physical mapping of genes on chromosomes, gene replication and collinearity analysis

The start and end locations of all genes on each chromosome were obtained from the potato database (), and the physical location and chromosome division information of *StCCR* gene of Atlantic potato were obtained according to the position of 48 chromosomes. The collinearity analysis of *StCCRs* was performed using MCScanX (Wang et al., 2012). Possible segment duplication and tandem duplication events were defined based on the physical location of the chromosomes of genes (Panchy et al., 2016), and the ratio of non-synonymous substitution to synonymous substitution (Ka/Ks) of duplicate gene pairs was determined by KaKs_Calculator (Zhang et al., 2006). Dual Synteny Plot in TBtools was used to analyze the collinearity of *StCCRs* with other species (Arabidopsis, tobacco, tomato) (Tang et al., 2008). The genomes of species files and annotations, respectively in the Ensembl the Plants (https://plants.ensembl.org/index.html) and an Eggplant Genome The Database (http://eggplant.kazusa.or.jp/index.html) to download. TBtools software was used to visualize the results (Chen et al., 2020).

### 2.3 Exon-intron, conserved motif analysis

Download the GFF file of the potato gene structure from the potato database (). The *StCCR* gene structure (exon-intron) was defined by TBtools software, and the conserved motif of StCCR protein was obtained from the online MEME program (https://meme-suite.org/) (Bailey et al., 2009). Use the following parameters: minimum width set to 6 bp, maximum width set to 25 bp, and maximum number of motifs set to 10. The structure and conserved domain of the *StCCR* gene were visualized by TBtools.

### 2.4 Multiple sequence alignment and phylogenetic analysis

In order to explore the evolutionary relationship of *CCR* gene family, multi-sequence comparison of CCR and other proteins of potato and *Arabidopsis thaliana* was performed using MEGA 7 (Sudhir et al., 2016) software, and the phylogenetic tree was constructed by Neighbor-joining method. using the maximum likelihood (ML) with 1000 bootstraps, other parameters default, to use online software Evolview v (https://www.evolgenius.info/evolview) to optimize the evolutionary tree.

### 2.5 Analysis of cis-acting components

The structure and conserved domain of *StCCR* gene were visualized by TBtools. The upstream 2000 bp sequence of the start codon of each *StCCR* gene was obtained from the potato genome. Through Plant CARE database (https://bioinformatics.PSB.Ugent.Be/webtools/plantcare/HTML/) of *StCCR* gene promoter sequence of cis element for on-line retrieval (Lescot, 2002).

### 2.6 Plant materials and stress treatment

The potatoes used in this study were of Atlantic variety. The original seed potatoes were provided by the College of Life Sciences of Northeast Agricultural University in China. They were planted in planting pots containing soil and vermiculite (in a ratio of 1:1) (dimensions: 40cm * 15cm * 15cm), and then placed in a laboratory incubator (temperature 22°C, humidity 60%, light cycle 16/8 hours, light intensity 10000 lx) for growth, with normal water and fertilizer management. To clarify the expression patterns of the *StCCRs* gene in different tissues of potatoes, as well as the expression patterns under various abiotic stresses and exogenous hormone stimulation, potato plants that had been growing normally for 30 days after emergence, with similar growth conditions, were selected. Samples were taken from the roots, above-ground stems, and leaves, wrapped in tin foil, and rapidly frozen in liquid nitrogen. They were then stored at -80°C for future use. According to researcher (Guan et al., 2020; Chang et al., 2024; Shi et al., 2025), taking the normally growing plants as the control group, the normal potato plants that had been growing normally for 30 days, those with good growth, and those with similar growth conditions were respectively subjected to high-temperature (42°C), low-temperature (4°C), salt (250 mmol/L NaCl), and drought (20% PEG6000) treatments. Compared with distilled water spray, leaves were sprayed with indole acetic acid (10 μmol/L IAA), gibberellin (50 μmol/L GA3) and abolic acid solution (10 μmol/L ABA), and then placed in a light incubator for culture. After treatment for 0,2,4,8,12,24 hours, samples were collected from leaves. Wrap with tin foil, freeze liquid nitrogen and store in a -80°C refrigerator for later use. All samples were taken for 3 replicates.

### 2.7 Real-time quantitative PCR

BIOER’s LineGene 9620 real-time fluorescent quantitative PCR system was used to perform qRT-PCR analysis on 10 genes. Q-StCCR Primer sequence (**Supplementary Table S9**) was designed by prime-blast (Ye et al., 2012). Total RNA was extracted from each collected sample using the Total RNA Rapid Extraction kit (ER501-01, TransGen Biotech, Beijing, China). and reverse-transcribed to cDNA using TransScript^®^One-Step gDNA Removal and cDNA Synthesis SuperMix (AT311, TransGen Biotech, Beijing, China). qRT-PCR was performed using ChamQ Universal SYBR qPCR Master Mix (Q711, Vazyme, Nanjing, China) kit. β-action was used as the internal reference gene (Tang et al., 2017), and three independent and repeated data were calculated by 2−^ΔΔCt^ method (Bustin et al., 2009). Analysis of variance (ANOVA) and drawing of gene expression heat maps were performed using GraphPad Prism 9 and TBtools.

### 2.8 Cloning of CDS sequence of *StCCR6* gene, construction of fusion expression vector and instantaneous expression

Potato leaves were taken, total RNA was extracted and its integrity was detected. cDNA synthesis was performed using a reverse transcription kit as the template for reverse transcription. The *StCCR6* gene sequence in potatoes was amplified by using the SnapGene software to design the primer StCCR6-1F/R, and it was cloned into the transient expression vector pCAMBIA2300-*StCCR6*-eGFP. The method of transient tobacco infection was referred to Mo et al. (2023). The localization of *StCCR6*-eGFP fluorescence signals was then observed using a laser confocal microscope (TCSSP8, Carl Zeiss, Germany).

### 2.9 Construction of *StCCR6* silent vector and plant infection

Design primers will be in the SGN - VIGS (https://vigs.solgenomics.net/) online design *StCCR6* best silent loci (VIGS*StCCR6*) amplification on pTRV2 carrier. The plasmids pTRV1, pTRV2, pTRV2-*PDS* and pTRV2-*StCCR6* were transferred into Agrobacterium GV3101 together. The resuspended pTRV2, pTRV2-*PDS* and pTRV2-*StCCR6* were respectively mixed with pTRV1 in equal volumes, and then left to stand in the dark at room temperature for 4 hours to obtain the TRV infection solution. VIGS transient infection was carried out on the leaves of 4-week-old potatoes. During the injection process, the main veins of the potatoes were avoided. After dark incubation for 2 days, they were transferred to the artificial culture room for normal management. The photobleaching symptoms of potato leaves after being treated with pTRV2-*PDS* infection were observed to confirm the successful infection. The *StCCR6* gene-silenced plants were identified by qPCR method.

### 2.10 Observation by projection electron microscopy

1. Materials: For plants that have been growing normally for 30 days, take the above-ground stem tissue from the same part and trim it into long strips that are 3 mm long and 1 mm wide. 2. Fixation: Pre-fixation: Fixation with 2.5% glutaraldehyde fixative for 2 hours; Rinse: Rinse 3 times with 0.1M phosphoric acid buffer solution (pH=6.8), each time for 15 minutes; Post-fixation: Fixation with 1% osmium acid fixative; Rinse: Rinse three times with 0.1M phosphate buffer solution (pH=6.8), each time for 15 minutes. 3. Dehydration: Stay in 50% ethanol for 15 minutes; Stay in 75% ethanol for 15 minutes; Stay in 90% ethanol for 15 minutes; Stay in 100% ethanol for 20 minutes; 100% ethanol +100% acetone = 1:1 Stay for 15 minutes; Stay in 50% acetone for 5 minutes. 4. Immersion: Pure acetone + embedding solution (1:1) for 1 day; Pure acetone + embedding solution (1:2) retention for 1 day; Pure acetone + embedding solution (1:3) retention for 1 day. 5. Embedding. 6. Aggregation: 3 days. 7. Repair the block. 8. Slicing with an ultra-thin slicer. 9. Electron microscope observation. The transmission electron microscope of Hitachi Scientific Instruments Beijing Co., LTD., model HT7800, has a high voltage of 80kV and magnifications of 2000, 5000 and 10000 times respectively. The thickness of the cell wall was measured using ImageJ.

### 2.11 Bacterial inoculation treatment and disease resistance verification of potato leaves

The *in vitro* leaf inoculation method was adopted. Sterile filter paper was placed in the culture dish and it was soaked with sterile water. Excessive water can easily cause leaf rot. The lint-free cotton is soaked and then wrapped around the petioles to retain moisture. Inoculate 20 μl of the bacterial liquid of bacterial blight (*Ralstonia solanacearum*, RS) (OD_600_ = 0.8) with a needle-free syringe at 1/4 of the surface of isolated leaves of healthy and disease-free silent and wild-type potatoes to allow the bacterial liquid to spread along the veins. Seal the petri dish and maintain humidity. Cultivate under room temperature and 16 h/d light conditions. The leaf conditions were recorded at 1, 2, 3, 4 and 5 days after pathogen treatment. The area of the lesion was statistically analyzed using ImageJ.

The overexpressed potato and wild-type potato leaves at 0 and 5 days after inoculation with the pathogen were used as materials. The contents of hydrogen peroxide (H_2_O_2_), superoxide anion (O2-), malondialdehyde (MDA), as well as the activities of superoxide dismutase (SOD), peroxidase (POD), and catalase (CAT) were determined according to the specific steps and calculation methods provided in the kit manual of Suzhou Komeng Biotechnology Co., Ltd., and the kit was used for the measurement. Three independent and repeated data were analyzed using GraphPad Prism 9 for variance analysis (ANOVA).

## 3 Results

### 3.1 Genome-wide identification and molecular characterization of cinnamyl coA reductase from Atlantic potato

**Figure 1:**
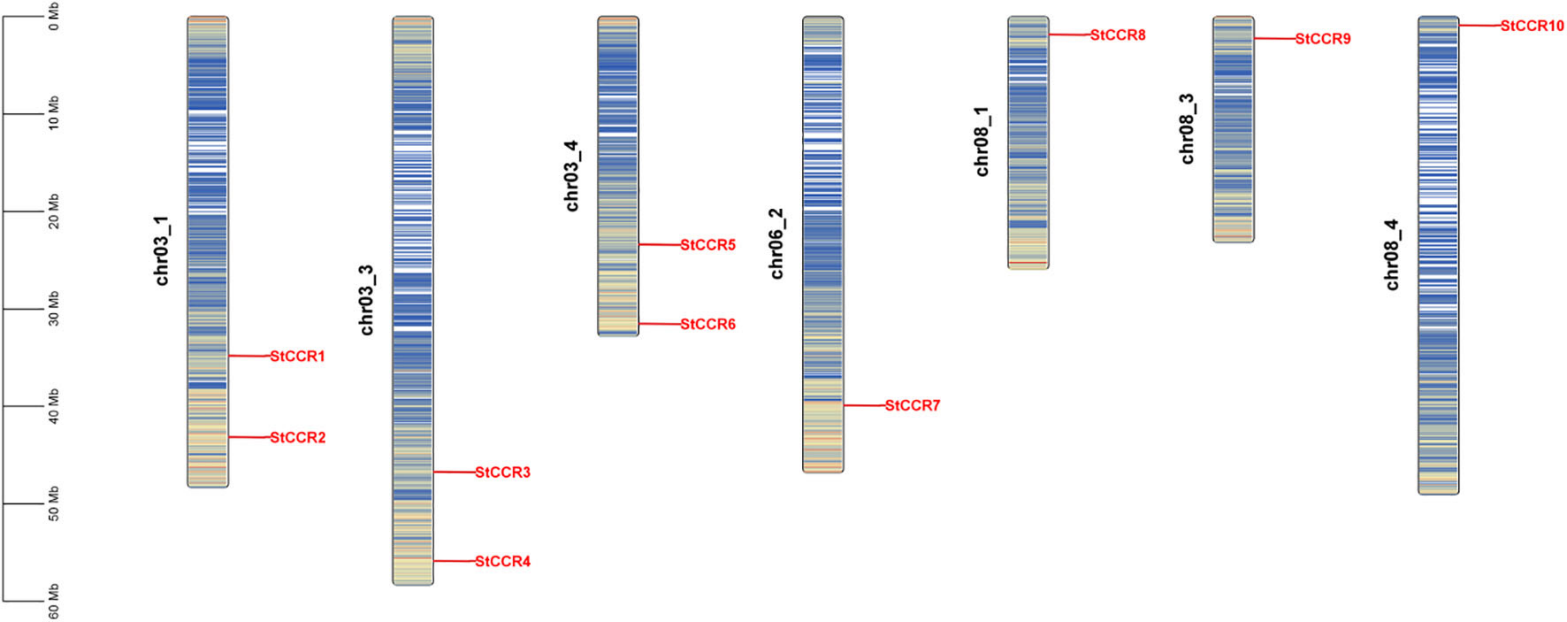
Chromosomal distribution of *StCCR* genes.

Systematic genome-wide identification revealed 10 *StCCR* genes in *Solanum tuberosum* L. (Atlantic variety), phylogenetically classified and renamed as *StCCR1*-*StCCR10* based on chromosomal localization (**Supplementary Table S1**). The encoded proteins exhibited conserved physicochemical properties: polypeptide lengths ranged from 206 (*StCCR8*) to 354 amino acids (*StCCR4*), with molecular weights spanning 34.5 kDa (*StCCR9*) to 39.4 kDa (*StCCR4*). Theoretical isoelectric points (pI) varied between 5.27 (*StCCR3*) and 7.02 (*StCCR7*), while aliphatic indices (77.01–95.29) and the GRAVY (Grand Average of Hydropathy) value of StCCRs ranges from -0.289 (*StCCR1*) to -0.046 (*StCCR9*). The instability index of *StCCRs* ranges from 23.32 to 37.04 (> 40 indicates protein instability, < 40 indicates protein stability), and both are stable proteins. Subcellular prediction results showed that *StCCRs* were located in cytoplasm (3) and chloroplasts (7), respectively. In order to reveal the distribution of CCR gene, we compared its sequence with potato genome data and used genomic localization information for chromosome mapping. As shown in **Figure 1**, a total of 10 *StCCRs* genes were unevenly distributed on 7 chromosomes of potato. Chromosomes chr3_1, chr03_3, chr03_4 contain two *StCCR* genes, while chromosomes chr06_2, chr8_1, chr08_3, ch8_4 contain only one *StCCR* gene. Chromosomal ideograms are scaled in megabases (Mb, left axis). Gene loci (red labels) denote physical positions of *StCCR* members across seven chromosomes. Gradient blue shading illustrates regional gene density, with darker tones corresponding to higher gene concentrations. Chromosome numbers are annotated adjacent to each ideogram.

### 3.2 Duplication analysis of *StCCRs* in Atlantic potato

**Figure 2:**
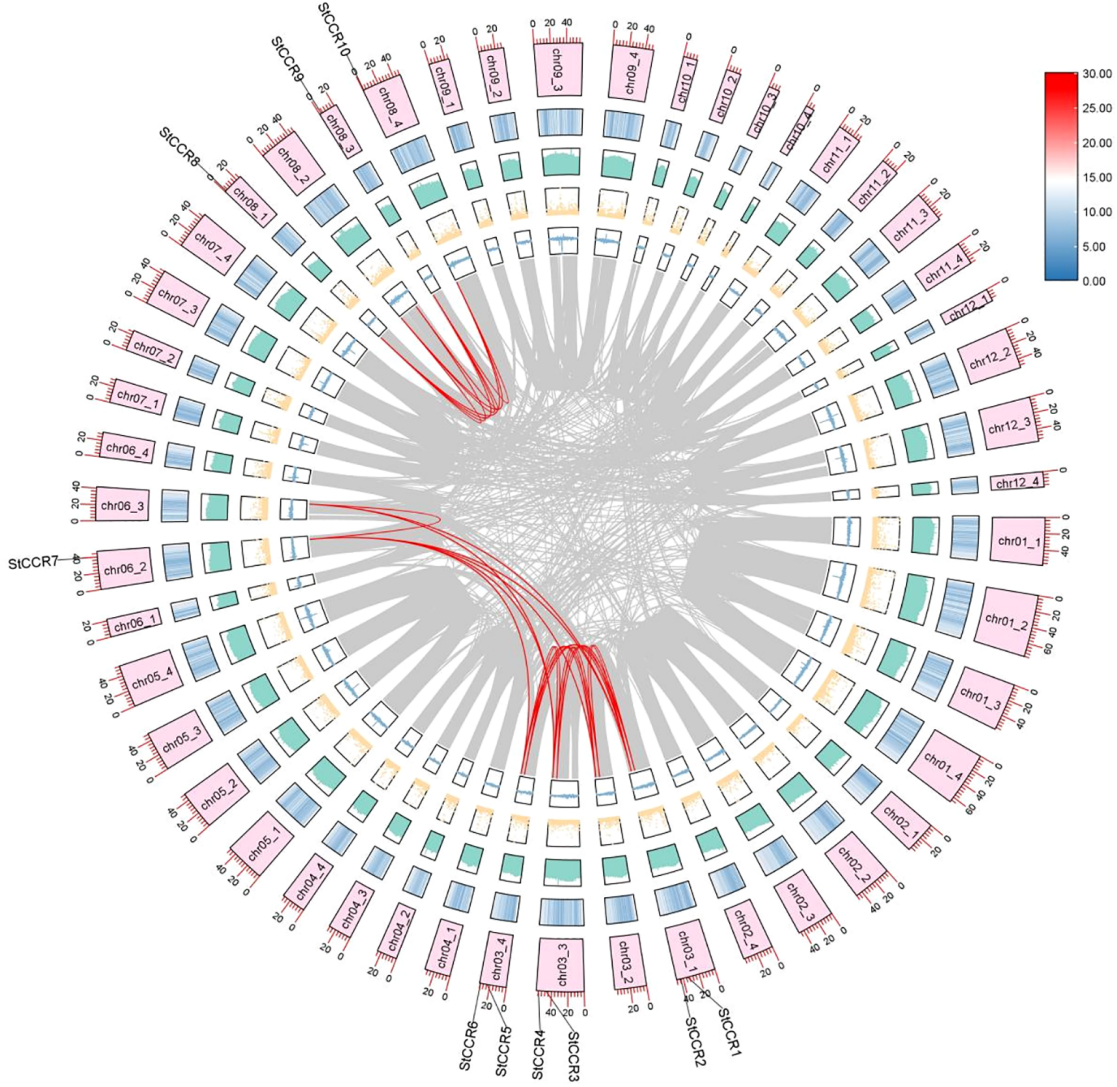
Synteny analysis of *StCCR* genes in the potato genome.

Collinear analysis was performed on these identified *StCCR* genes, and it was found that 10 *StCCR* genes participated in 12 duplicate gene pairs (**Figure 2**; **Supplementary Table S2**). Notably, *StCCR2*, *StCCR4*, *StCCR6*, and *StCCR7* each participated in three duplication events, whereas other members engaged in two events. These results indicate that *StCCRs* is mainly amplified by fragment repetition during the evolution of Atlantic potato. The nonsynonymous substitution replacement rate (Ka) and synonymous replacement rate (Ks) are the basis for evaluating whether collinear genes are subjected to selection pressure. Ka/Ks value 1 indicates natural selection, Ka/Ks < 1 indicates purification selection, and Ka/Ks > 1 indicates positive selection (Zhang et al., 2006). Selection pressure analysis (**Supplementary Table S3**) demonstrated Ka/Ks ratios ranging from 0.0347 to 0.8897 across duplicated pairs, consistent with strong purifying selection (Ka/Ks < 1). This evolutionary constraint indicates functional conservation within the *StCCR* family, though retained paralogs may exhibit conditional redundancy.*StCCR* gene synonym substitution is more favorable for potato evolution, and the low Ka/Ks ratio indicates that non-synonym mutations are largely excluded, a phenomenon commonly observed in functionally important and conserved genes, such as genes encoding metabolically critical enzymes or structural proteins. Depicted are concentric tracks representing genomic features (outer to inner): chromosome ideograms scaled in megabases (Mb), gene density heatmap (red = high density, blue = low), GC content histogram (height proportional to GC ratio), N-content dot plot (dot density reflecting unknown base frequency), and GC skew bars (blue = guanine excess, yellow = cytosine excess). Syntenic relationships among *StCCR* paralogs are highlighted by red connecting lines.

### 3.3 Collinear analysis of *StCCR* genes

**Figure 3:**
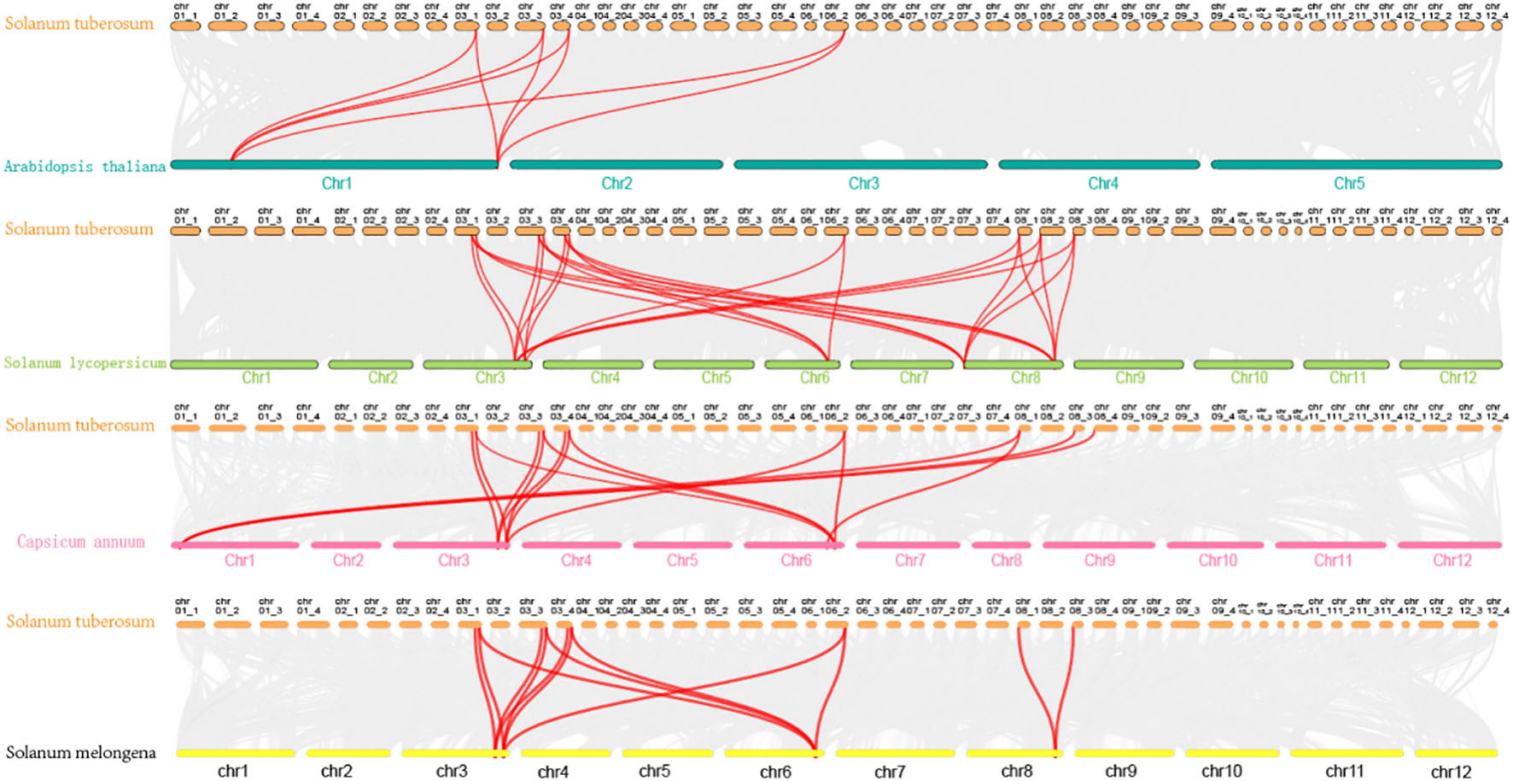
Homology analysis of CCR genes between Atlantic potato and four representative species.

To investigate genomic expansion mechanisms and evolutionary relationships of *StCCRs*, we performed collinearity analysis between potato and four representative species (*Arabidopsis thaliana*, *Solanum lycopersicum*, *Capsicum annuum*, and *Solanum melongena*) using potato *StCCR* sequences as anchors (**Figure 3**). Comparative synteny revealed stronger conservation within Solanaceae, with potato-tomato collinear pairs (26 pairs) significantly exceeding those of potato-pepper (15 pairs), potato-eggplant (13 pairs), and potato-Arabidopsis (8 pairs). Chromosomal distribution analysis identified conserved syntenic blocks predominantly localized to chr3, chr6, and chr8 in Solanaceae species, while potato-Arabidopsis collinear pairs clustered on chr1, chr3, and chr6 (**Supplementary Table S4**). Gray lines indicate homologous genomic blocks in potato relative to other species. MCscanX was employed to identify collinear relationships between potato *StCCRs* and homologous genes in *Arabidopsis thaliana*, tomato (*Solanum lycopersicum*), pepper (*Capsicum annuum*), and eggplant (*Solanum melongena*), with syntenic gene pairs highlighted by red connectors. The analysis showed that these homologous gene pairs may have existed before the differentiation of ancestral lineages, collinearity retained genes are conserved and may have core functions, and they exist stably in collinearity blocks across species, which also implies that the Atlantic potato *StCCRs* gene family may also expand and evolve through gene replication.

### 3.4 Phylogenetic analysis and structural characterization of *StCCRs*

**Figure 4:**
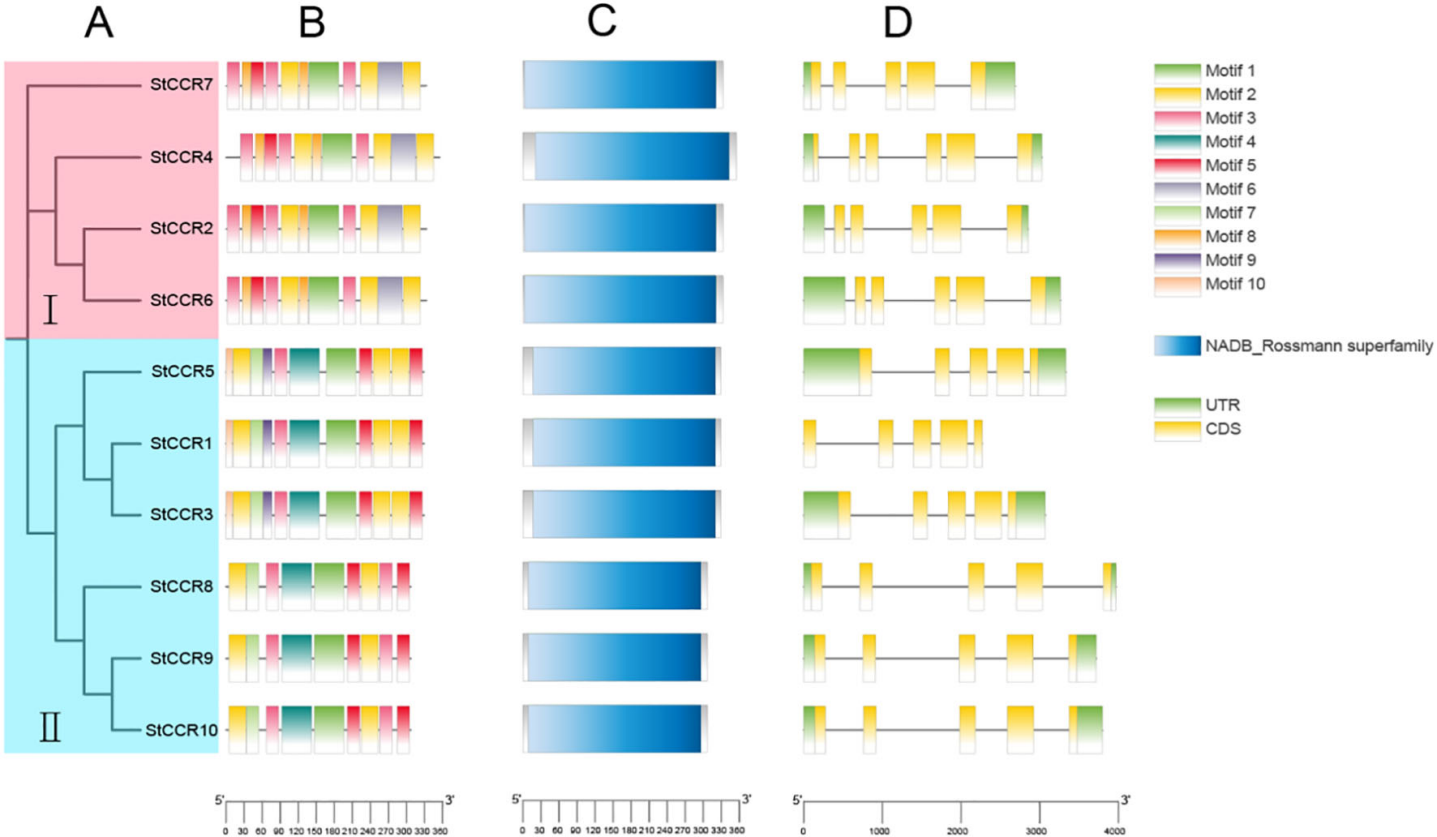
Phylogenetic and structural characterization of *StCCR* genes. **(A)** Neighbor-joining phylogenetic tree of *StCCR* proteins; **(B)** Distribution patterns of putative conserved motifs identified in *StCCRs*; **(C)** Predicted NADB_Rossmann superfamily domains in *StCCR* proteins; **(D)** Exon-intron organization of *StCCR* genes across two subfamilies.

To elucidate evolutionary relationships among potato *CCR* members, a neighbor-joining (NJ) phylogenetic tree was constructed based on nucleotide sequences. The 10 *StCCRs* clustered into two subfamilies: Subfamily I contained four genes, while Subfamily II comprised six members (**Figure 4A**). Conserved motifs in StCCR proteins were predicted using MEME, identifying 10 distinct motifs (**Figure 4B**; **Supplementary Table S5**). motif composition and distribution are relatively conservative among members of the same subgroup, and the type and number of motifs in the same subgroup are similar. All proteins contain motif1, 2, 3, and 5 motifs, among which there are conserved motifs specific to CCR proteins, and these motifs are also conserved during the evolution of potato CCR, which may be necessary for maintaining the function of CCR proteins. CCR members clustered in the same branch have similar motif composition and motif position. For example, motif6 and 8 appear in subfamily I, motif4 and 7 appear in subfamily II, and motif9 and 10 are specific to *StCCR1*, *StCCR3* and *StCCR5*. Domain architecture analysis via NCBI CDD revealed that all *StCCRs* belong to the NADB_Rossmann superfamily, confirming conserved catalytic domains (**Figure 4C**). Gene structure comparisons demonstrated distinct intron-exon organizations: Subfamily II members contained five exons and four introns, while Subfamily I exhibited six exons and five introns except for *StCCR7* (**Figure 4D**). Sequence similarity and high similarity in intron-exon structure among members of the two subfamily suggest that the potato cinnamyl CoA reductase gene may have undergone a gene replication event during evolution.

### 3.5 Phylogenetic analysis of StCCRs

**Figure 5:**
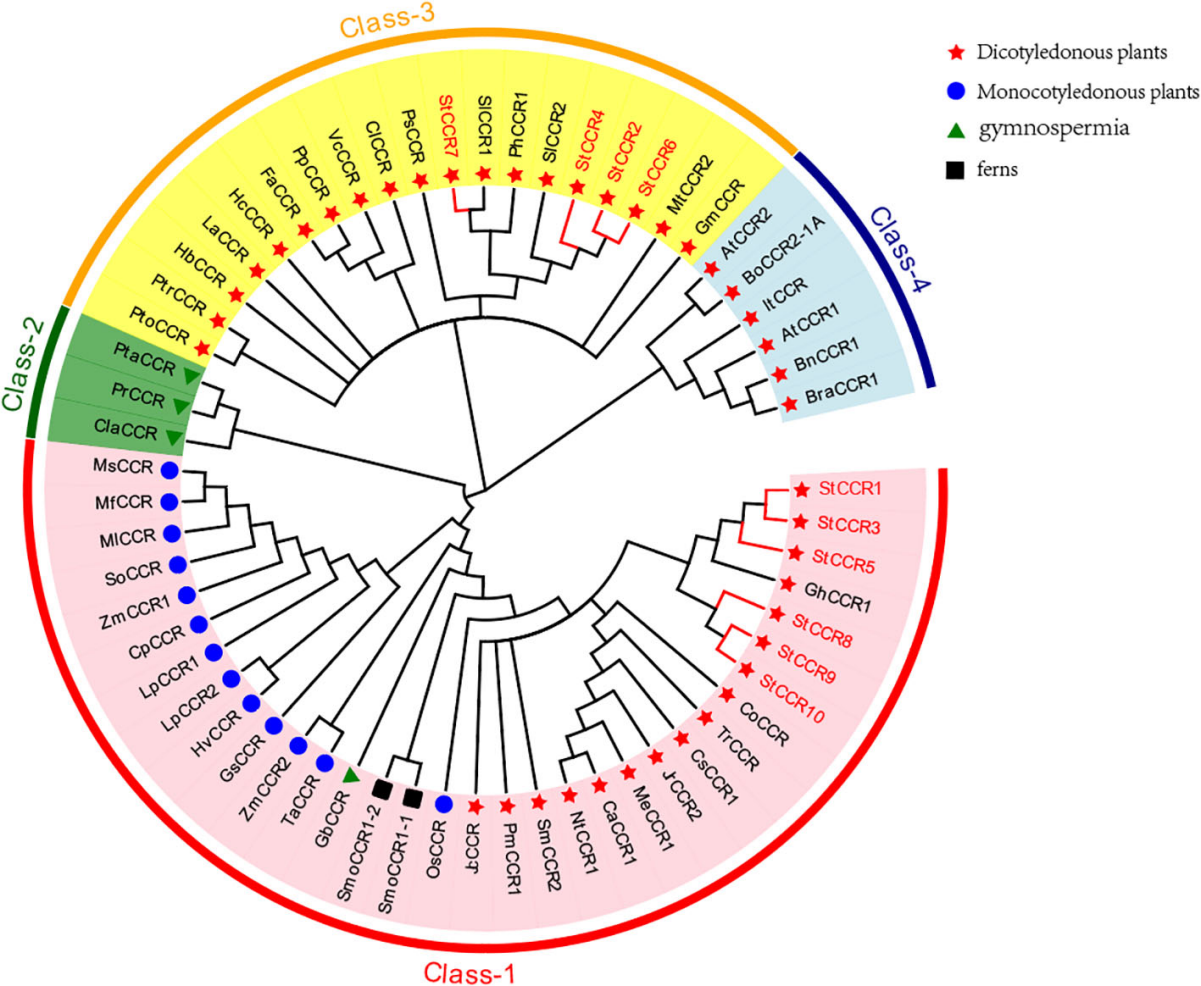
Phylogenetic reconstruction of CCR gene relationships across plant taxa.

To elucidate evolutionary relationships within the CCR family, we constructed a phylogenetic tree using 61 CCR protein sequences from diverse species, including 10 *StCCRs* from Atlantic potato (**Figure 5**). The analysis revealed four distinct clades: Clade I contained 33 CCRs from monocots, dicots, gymnosperms, and ferns, incorporating six *StCCRs* from Subfamily II (*StCCR1/3/5/8/9/10*). Clade II exclusively comprised three gymnosperm CCRs. Clade III included 19 CCRs, notably containing four *StCCRs* from Subfamily I (*StCCR2/4/6/7*) that clustered closely with two Solanaceae homologs (*SlCCRs* and *PhCCR1*) and showed evolutionary proximity to Arabidopsis CCRs in Clade IV. We speculate that these 4 *StCCR* proteins may be related to the real CCR function, and whether the genes of *StCCR* subfamily II have enzyme activity needs to be further proved by subsequent studies and experiments. Taxonomic groups are denoted by distinct markers: dicots (red triangles), monocots (blue circles), gymnosperms (green triangles), and ferns (black squares). Clade-specific coloration indicates evolutionary groupings: Clade I (red), Clade II (green), Clade III (yellow), and Clade IV (blue). Complete species designations and corresponding sequence accession numbers are provided in **Supplementary Table S6**.

**Figure 6:**
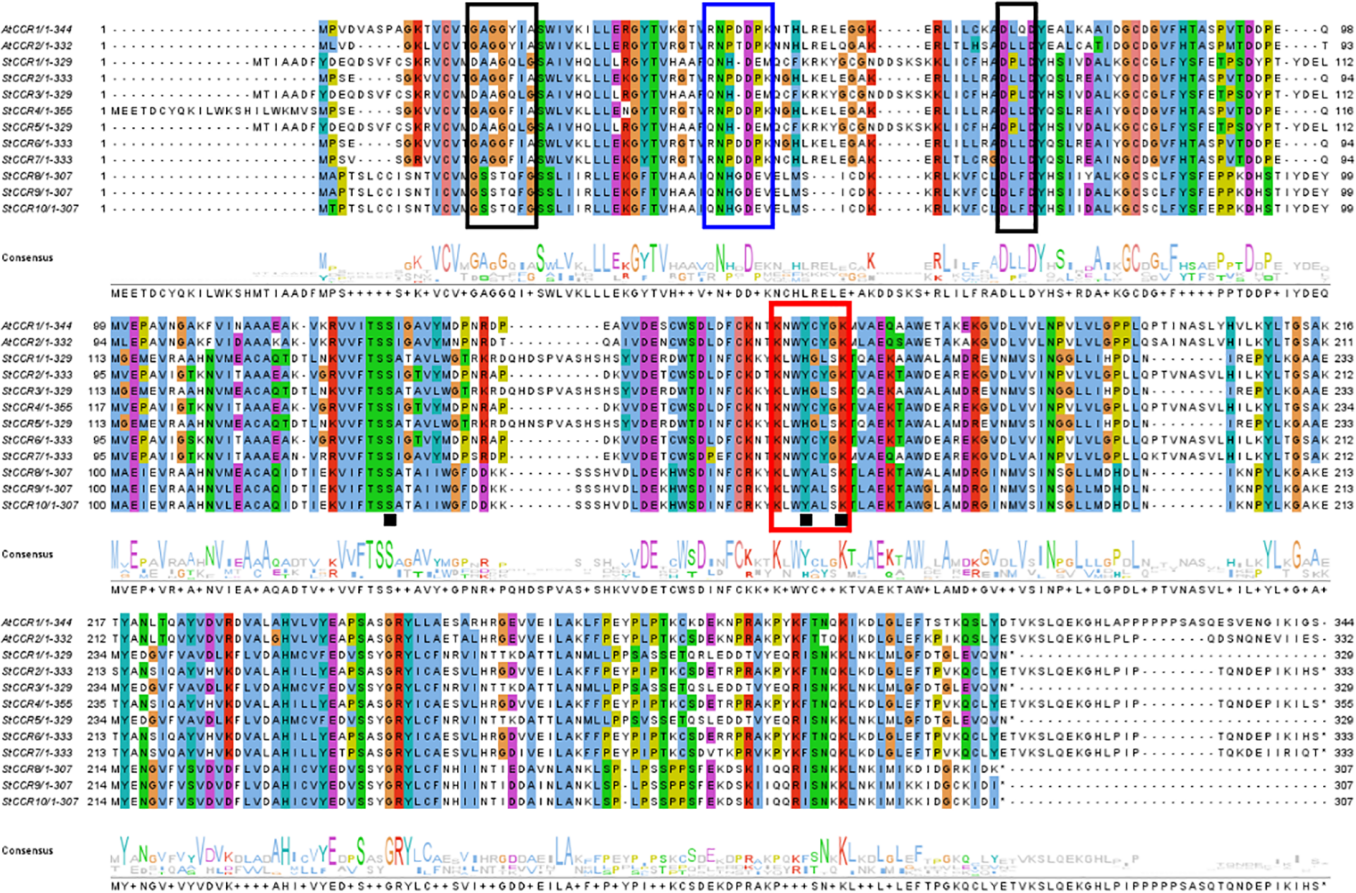
Sequence alignment of StCCR proteins with two *Arabidopsis* CCR homologs (AtCCRs).

A conserved CCR signature motif related to NADP (H) binding and substrate catalysis was found in the amino acid sequence of most plant CCR proteins. This region can form a β-α-β structure in the secondary structure, and it is speculated that it may be the catalytic site of CCR, or the binding region of its cofactor NADPH, especially the two lysine residues on it may directly bind to the substrate. Conserved motifs G-X-X-G-X-A and D-X-X-D have been reported to be involved in NAD (P) binding and adenine pocket stable binding. In addition, NADP+ specific motifs R (X) 5K have been identified, which is A key structure that distinguishes CCR from other NAD (H) dependent SDRS (Pan et al., 2014; Chao et al., 2017, 2019).The comparison between two AtCCR proteins and 10 StCCR proteins showed (**Figure 6**) that both AtCCR proteins and StCCR subfamily I proteins had a KNWYCYGK conserved motif. However, changes in the conserved motifs of subfamily II May affect protein binding to substrates and catalytic activity. This is similar to the situation of rice and maize (Joël Piquemal et al., 2010; Bai et al., 2003). The comparison between AtCCR/StCCR proteins showed that some of them had the NAD (P) binding motif GXXGXXA/G. The catalytic triplet Ser-Tyr-Lys has been confirmed as an active SDR residue (Chao et al., 2017), and the corresponding conserved motif Y-X-X-X-K has been identified in all AtCCR/StCCR proteins. The diversity of conserved motifs in StCCR proteins suggests that they may have different activities and biological functions. Functionally critical motifs are annotated: CCR signature motif (red boxes), NAD (P)-binding domains (black boxes), NADP specificity determinant R(X)5K (blue boxes), and catalytic triad residues Ser-Tyr-Lys (black squares).

### 3.6 Cis-regulatory element analysis of *StCCRs*

**Figure 7:**
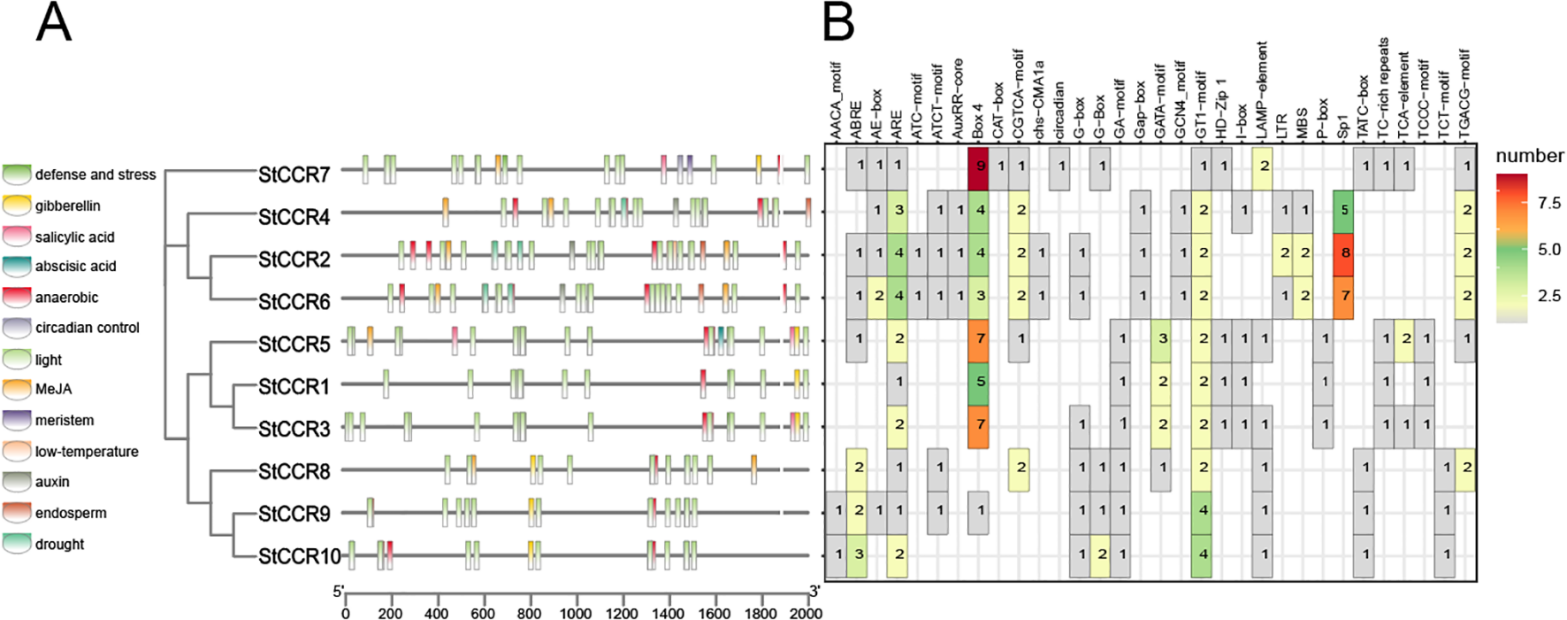
Putative cis-element and transcription factor binding sites in the promoter region of *StCCR* genes. **(A)** represent the different types of cis-acting elements and their positions in each StCCR genes; **(B)** shows the number of cis-acting components.

Promoter cis-elements play a key role in the initiation of gene expression. In order to further investigate the response of *StCCR* gene family to abiotic stress, we analyzed the promoter cis-acting elements of *StCCR* family members (Oudelaar and Higgs, 2021). Through in-depth analysis of 2000 bp sequences upstream of each gene translation initiation site, it was found that promoters of *StCCR* gene family members contain multiple cis-acting elements. The results (**Figure 7**) showed that the cis-acting elements in the promoter region of *StCCR* gene involved in stress response were mainly divided into photoresponsive elements, hormone-responsive elements, environmental stress-related elements and site-binding elements. The colored squares in **Figure 7A** represent the different types of cis-acting elements and their positions in each *StCCR* genes; **Figure 7B** shows the number of cis-acting components. Three types of elements involved in plant hormones, light response and environmental stress response are particularly abundant. All *StCCR* promoter sequences contain ARE response elements and light response elements related to anaerobic induction (for example, GT1-motif and some genes contain a large number of Box-4 cis-acting elements). Among the elements responding to plant hormones, *StCCRs* may be involved in plant responses to plant hormones such as methyl jasmonate, salicylic acid, ABA, auxin and gibberellin. ABRE responsive elements, AuxRR-core, P-box, CGTCA-motif and TGACG-motif related to jasmonic acid reaction, and TCA-element involved in salicylic acid reaction also exist in some *StCCRs* gene promoters. Abiotic stress-related elements include LTR, MYB binding sites MBS, defense and stress-related regulatory elements (TC-rich repeats). *StCCR2*, *StCCR4* and *StCCR6* also have Sp1 elements related to cell growth and differentiation. These results suggest that the expression of *StCCRs* may be regulated by light stimulation, anaerobic induction, plant hormones and abiotic stress during potato growth, and play an important role in protecting the plant against stress.

### 3.7 Organ-specific expression patterns of *StCCRs*

**Figure 8:**
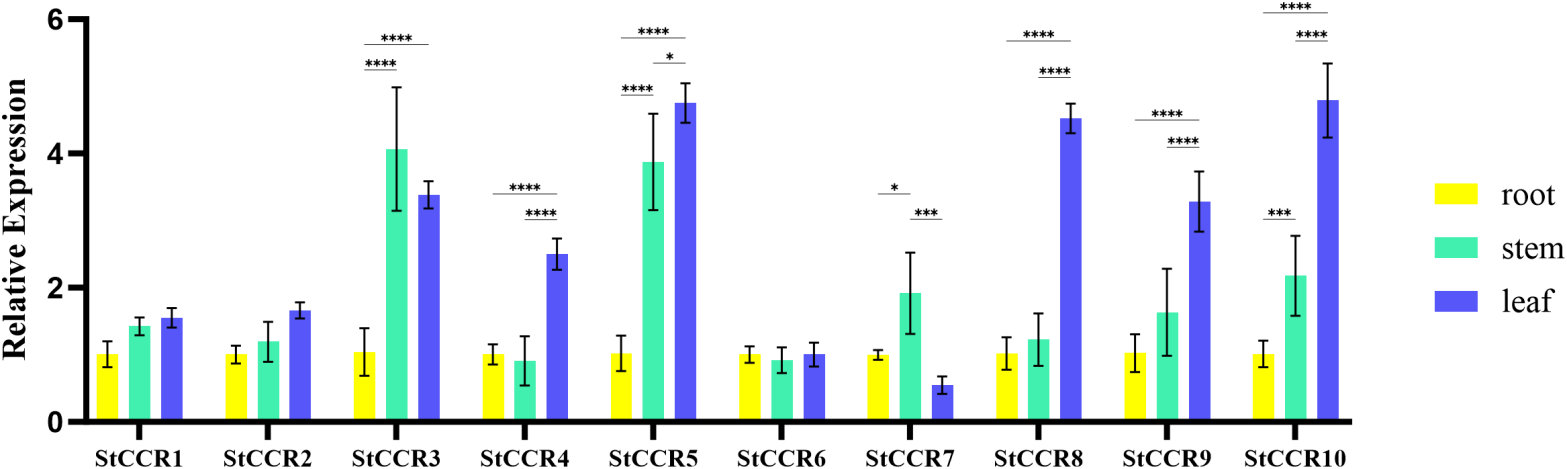
Relative expression levels of *CCR* gene in roots, stems and leaves of potato. The experimental data are presented as the mean ± standard error from at least three independent biological replicates. Stars indicate statistical significance by One-way ANOVA analysis: * p-value < 0.05, *** p-value < 0.001, **** p-value < 0.0001.

Gene expression serves as a critical determinant of gene function. To investigate the expression profiles of potato *CCR* genes across plant organs during growth stages, qRT-PCR was performed using the *β-actin* gene as an internal reference and root tissues as the baseline control (**Supplementary Table S7**). The organ-specific expression analysis (**Figure 8**) revealed that most *StCCRs* exhibited higher relative expression levels in stems and leaves compared to roots. Notably, Subfamily II members including *StCCR3*, *StCCR5*, *StCCR8*, and *StCCR10* demonstrated pronounced leaf-predominant expression, while *StCCR3* and *StCCR5* showed elevated expression in stems, all of which were higher than those of Subfamily I genes. The fact that the *CCR* genes were more highly expressed in the stems and leaves of potato plants than in the roots suggests that lignin biosynthesis is more active in these above-ground tissues. CCR is a key enzyme in the phenylpropanoid pathway that produces lignin, a structural compound that strengthens cell walls. In short, higher *CCR* expression in stems and leaves means that these tissues invest more in structural support and defense, possibly due to their exposure to mechanical stress, pathogens, or the need for rigidity for upright growth and photosynthesis. Moreover, different *StCCR* genes have different functions in various organs of potatoes.

### 3.8 Analysis of *StCCRs* gene expression patterns under different stress treatments

**Figure 9:**
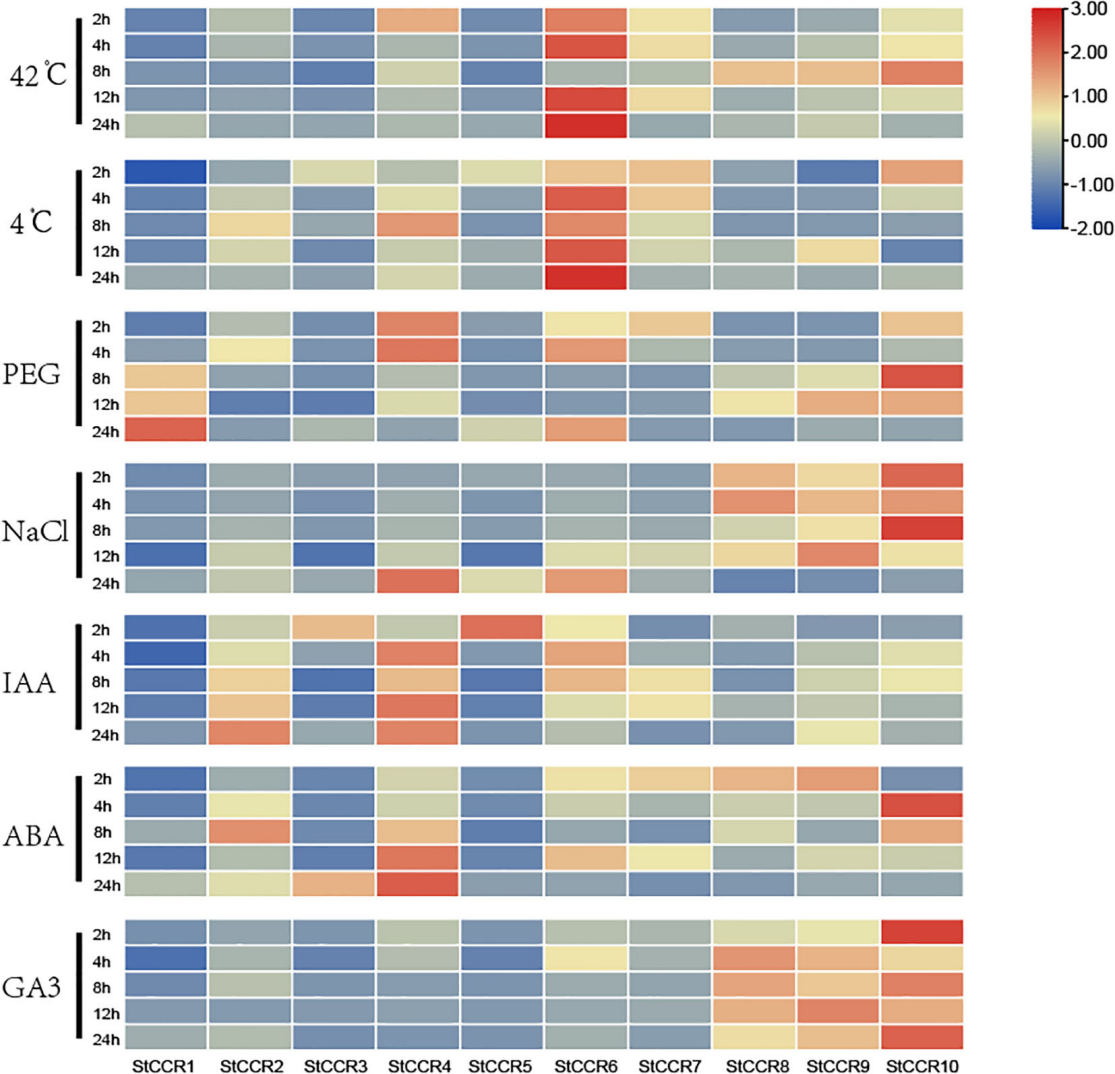
Heat maps of *CCR* gene expression profiles in potato under 7 different abiotic and hormonal stresses within 24 h. Abiotic stress includes high temperature, low temperature, mannitol, salt; Hormone treatments include IAA, ABA and GA3.

Abiotic stresses (drought, salinity, extreme temperatures) and hormonal stimuli constitute critical constraints on potato productivity. Building upon prior bioinformatics and cis-element analyses, we investigated *StCCR* responses to these challenges through qRT-PCR quantification under high-temperature (42°C), low-temperature (4°C), drought, salt, IAA, ABA, and GA3 treatments (**Supplementary Table S8**). All seven treatments significantly modulated CCR transcript levels within 24 hours (**Figure 9**). *StCCR1/3/5* exhibited basal expression across most conditions, while *StCCR4/6/10* demonstrated marked responsiveness. *StCCR2/4/6/7* are the same subfamily with close evolutionary relationship, and the gene expression levels and trends of each gene in each treatment are similar in each group. *StCCR8/9/10* as another group, the situation is similar. The expression patterns of the remaining genes were different due to different stress and genes. Thermal treatments elicited temporal-specific responses: *StCCR6* maintained sustained activation throughout 2–24 h exposure, and the relative expression levels of *StCCR8/9/10* at 42°C increased first and then decreased after 24 h treatment with *StCCR2/4/8* at 4°C. The relative expression levels of *StCCR2/4/7* at 42°C and *StCCR3/5/7/10* at 4°C for 24 h showed a trend of continuous decline. The expression of CCR gene decreased after treatment with low temperature for a certain period of time. We speculated that CCR gene in plants gradually decreased with low temperature to reduce lignin synthesis and accumulation, so that water in plant cells could flow out more easily, and the influence of ice crystals caused by low temperature on cell structure could be reduced. Under drought treatment, the relative expression of *StCCR1* continued to increase with the increase of treatment time, which was also one of the most significant responses of *StCCR1* in many treatments. The relative expression levels of *StCCR2/4/7* genes remained at a high level during 2–4 h, and then showed a downward trend, while the expression levels of *StCCR8/9/10* in group 2 increased first and then decreased. Under salt stress, the expression levels of *StCCR2/4/6* in subfamily I continued to increase and reached the highest level at 24 h, while in subfamily II, on the contrary, reached the lowest level at 24 h. Phytohormone responses revealed differential kinetics: IAA and ABA treatments upregulated both subclades, with *StCCR2/4* showing pronounced time-dependent amplification. GA3 activated Subfamily II members (*StCCR8/9/10*). These findings highlight functional diversification among *StCCRs*, with *StCCR4/6/10* emerging as key stress-responsive candidates through their consistent transcriptional alterations across multiple challenges.

### 3.9 Subcellular localization of *StCCR6*.

**Figure 10:**
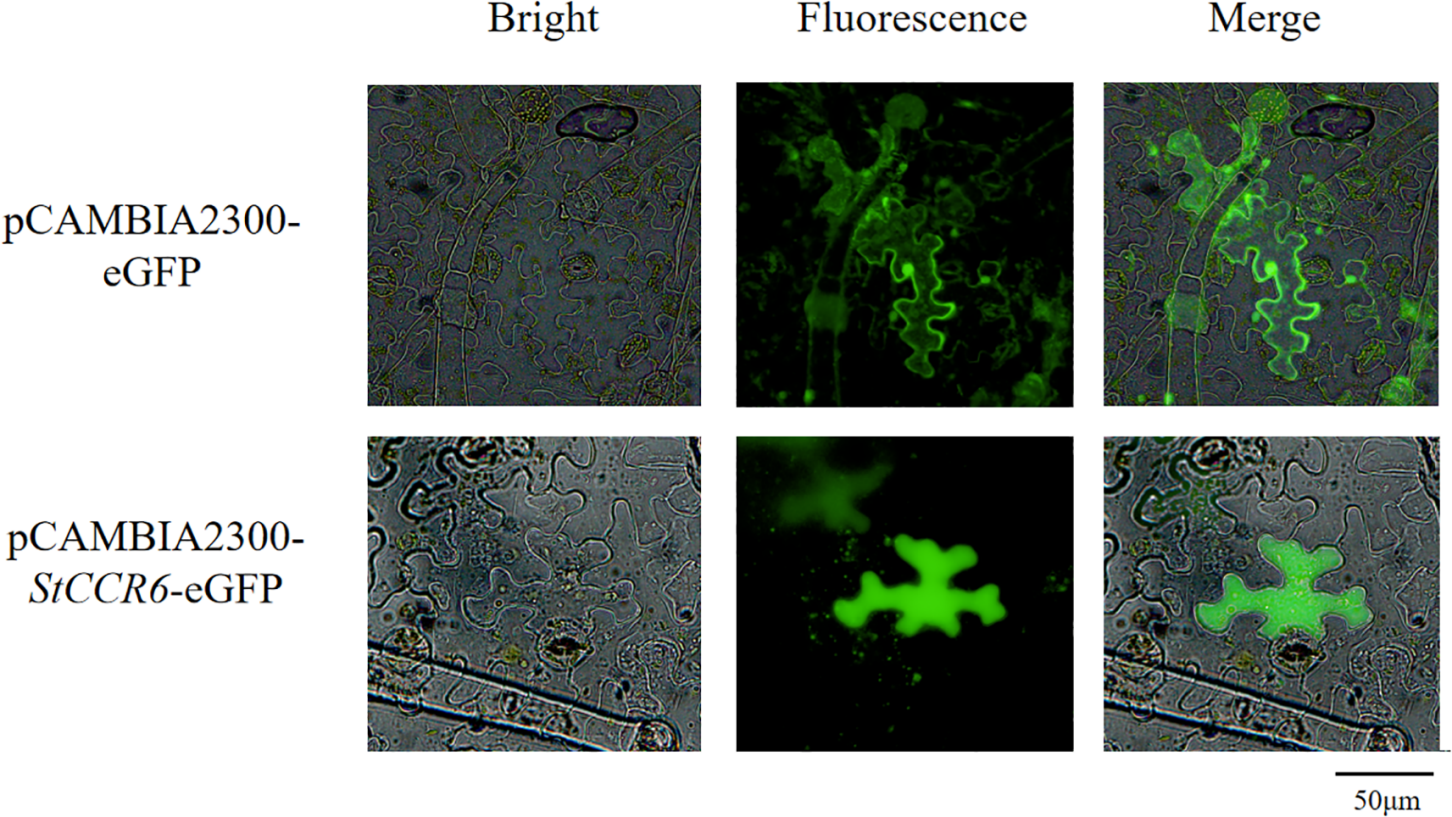
Subcellular localization of *StCCR6*.

To determine the subcellular localization of *StCCR6*, Agrobacterium-mediated transient transformation of Nicotiana benthamiana leaves was performed using the pCAMBIA2300-*StCCR6*-eGFP fusion construct, with the empty pCAMBIA2300-eGFP vector serving as the control. Transfected leaves were maintained at 26°C under low-light conditions for 48 h prior to fluorescence examination. The results (**Figure 10**) showed that the empty pCAMBIA2300-eGFP construct displayed green fluorescence signals in the nucleus, cytoplasm, and cell membrane. In contrast, GFP fluorescence of the fusion protein pCAMBIA2300-*StCCR6*-eGFP appears in the cytoplasm.

### 3.10 Silencing of potato *StCCR6* and functional analysis in lignin synthesis

**Figure 11:**
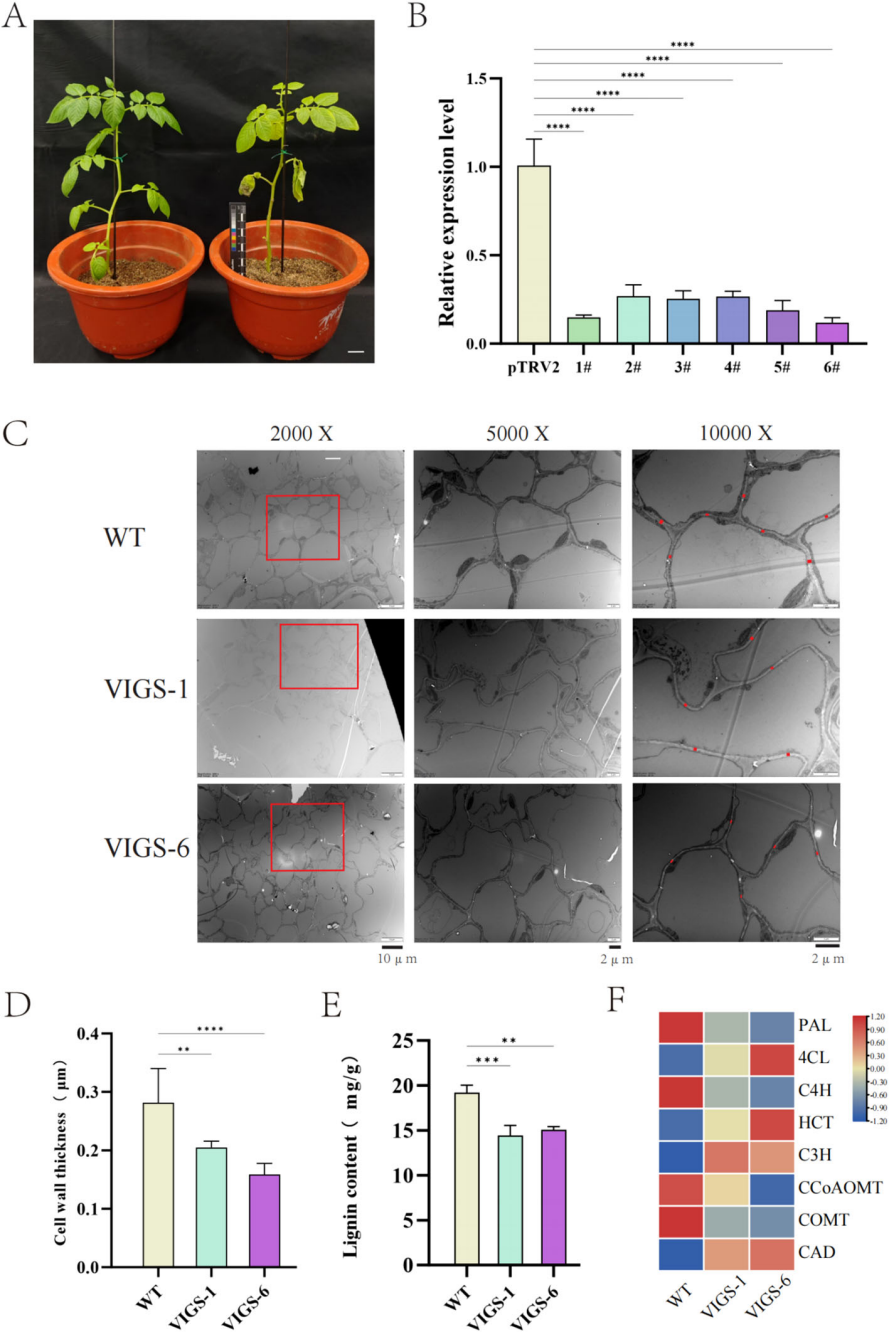
pTRV2-*StCCR6* silence and related data. **(A)** Silent phenotype. **(B)** Silence efficiency measurement. **(C)** Observation of the stem cross-sections of silent plants under projective electron microscopy. **(D)** Cell wall thickness statistics. **(E)** Determination of lignin content in silenced plants. **(F)** Relative expression of genes related to lignin biosynthesis. The experimental data are expressed as the mean ± standard error of at least three independent bioreplicates. Stars indicate statistical significance by One-way ANOVA analysis: ** p-value < 0.01, *** p-value < 0.001, **** p-value < 0.0001.

Bioinformatic analysis combined with qRT-PCR profiling identified differential expression patterns among *StCCR* subfamily members under various stress conditions, with *StCCR6* demonstrating particularly pronounced transcriptional regulation. To functionally characterize *StCCR6*, virus-induced gene silencing (VIGS) was implemented via Agrobacterium-mediated transformation using the tobacco rattle virus (TRV) vector system. **Figure 11A** illustrates experimental controls, with left panel showing asymptomatic pTRV2 empty vector plants and right panel displaying characteristic photobleaching in pTRV2-*PDS* positive controls (phytoene desaturase *PDS* serving as VIGS validation marker). The gene silencing efficiency of PTRV2-*StCCR6* detected by qRT-PCR was 85.1%, 73.3%, 74.7%, 73.7%, 81.2%, 88.1%, respectively, and the gene expression level was lower than that of pTRV2 (control) plants. Subsequently, strains 1# and 6# with high selection efficiency were selected for subsequent experimental research (**Figure 11B**) and named VIGS-1 and VIGS-6.

Transmission electron microscopy (TEM) analysis of stem cross-sections revealed ultrastructural differences between silenced and WT plants (**Figure 11C**). Quantitative measurements demonstrated 14.8% (VIGS-1: 0.2395 μm) and 25.1% (VIGS-6: 0.2105 μm) reductions in cell wall thickness relative to WT plants (0.281 μm) (**Figure 11D**). By further measuring the lignin content of WT plants and silent plants, it was found that the lignin content of WT plants was higher than that of silent plants (**Figure 11E**).

Transcriptional profiling of phenylpropanoid pathway genes (**Figure 11F**) revealed differential regulation in silenced plants: *StPAL*, *StC4H*, *StCCoAOMT*, and *StCOMT* exhibited elevated expression in WT plants, whereas *St4CL*, *StHCT*, *StC3H*, and *StCAD* showed reduced expression compared to silenced lines. These results suggest *StCCR6* modulates lignification through coordinated regulation of phenylpropanoid biosynthesis components.

### 3.11 Phenotype and ROS damage analysis of pTRV2-*StCCR6* under stress of RS bacteria

**Figure 12:**
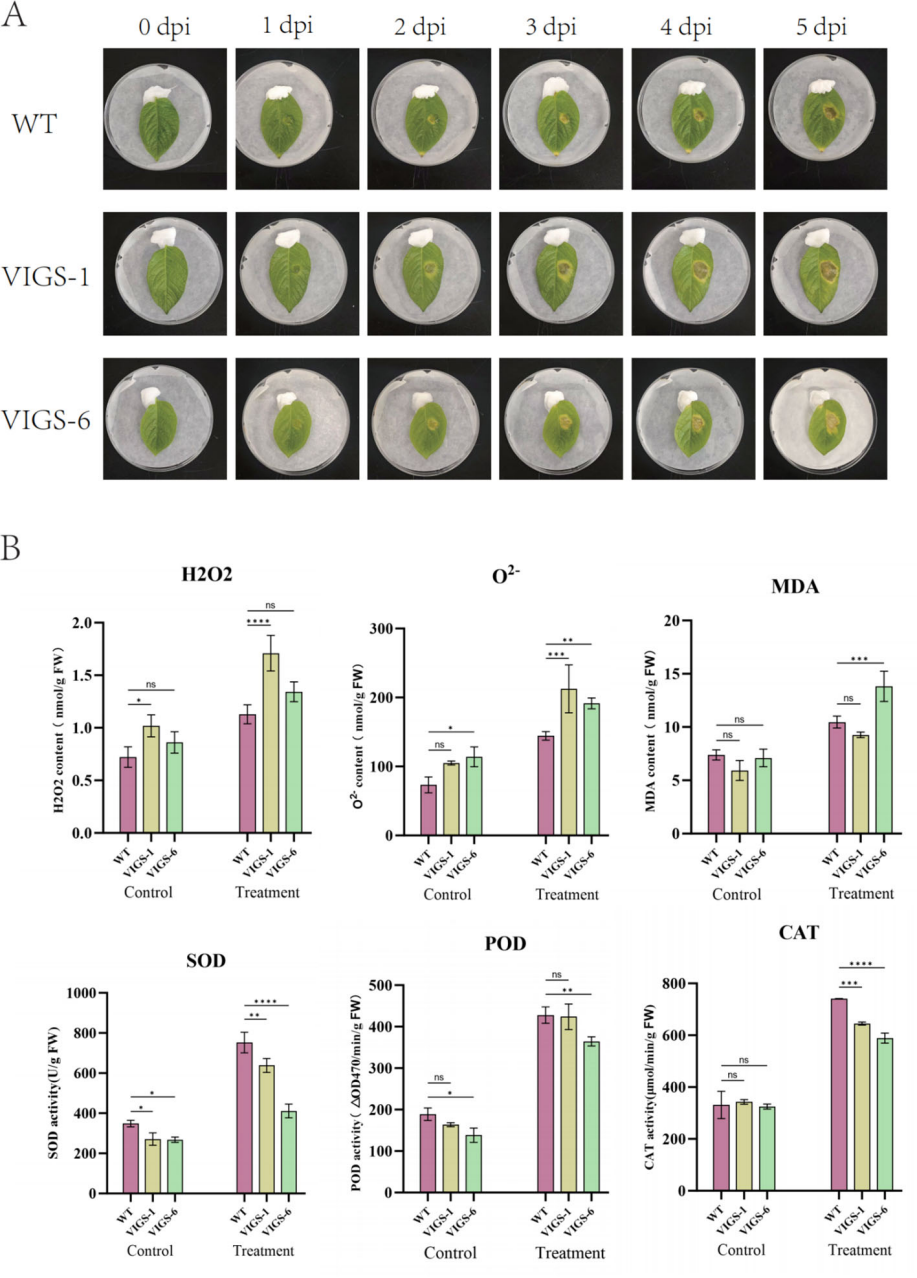
Disease resistance analysis of pTRV2-*StCCR6* and control plants under RS strain stress. **(A)** Phenotype observation after inoculation. **(B)** Antioxidant enzyme activity, including H_2_O_2_, O^2-^, MDA, SOD, POD, CAT, etc.; Control: leaves untreated; Treatment: Treatment of the leaves for 5 days. The experimental data are expressed as the mean ± standard error of at least three independent bioreplicates. Stars indicate statistical significance by One-way ANOVA analysis: *p-value < 0.05, **p-value < 0.01, ***p-value < 0.001, ****p-value < 0.0001.

Under adverse conditions, plants generate reactive oxygen species (ROS) that induce cellular toxicity, leading to structural damage and programmed cell death in plant tissues. Comparative analysis of 30-day-old *StCCR6*-silenced plants (pTRV2-*StCCR6*) and empty vector controls (pTRV2) revealed differential responses to bacterial challenge in controlled light conditions. **Figure 12A** demonstrates progressive leaf lesion development over a 5-day infection period, with pTRV2-*StCCR6* lines exhibiting significantly enhanced susceptibility compared to controls. Quantitative measurements showed RS-inoculated WT plants developed lesions averaging 0.918 cm^2^, while VIGS-1 and VIGS-6 silenced lines displayed 2.26-fold (2.075 cm^2^) and 2.02-fold (1.852 cm^2^) increases in necrotic area, respectively. These findings suggest *StCCR6* silencing compromises potato resistance to RS infection, exacerbating foliar damage severity.

In order to evaluate oxidative damage, the contents and activities of H_2_O_2_, O^2-^, SOD, POD, CAT and MDA in leaves of *StCCR6*-silenced plants and WT plants under RS stress were detected. The result is shown in **Figure 12B**. After inoculation, the accumulation of H_2_O_2_ and O^2-^ in potato leaf cells increased, and pTRV2-*StCCR6* was higher than that in control plants. MDA is the product of membrane lipid peroxidation, and its content indirectly reflects the degree of oxidative damage of cells. Before inoculation, there was no difference in the content of silent plants and WT plants, but the MDA content of plants after inoculation was accumulated. Among them, VIGS-6 was significantly increased compared with WT, and VIGS-1 was not different from WT. SOD, POD and CAT are important antioxidant enzymes in plants. There was no significant difference in physiological indexes of potato leaves before inoculation. Due to the stress of RS bacteria, the contents of SOD, POD and CAT of silent plants were lower than those of WT except POD of VIGS-1.

## 4 Discussion

The 10 *StCCR* gene family members identified in this study are close in number to those of *Arabidopsis thaliana* (11) (Lauvergeat et al., 2001), *Populus tomentosa* (11) (Chao et al., 2019), *Eucalyptus grandis* (10) (Carocha et al., 2015), and *Populus tomentosa* (9) (Shi et al., 2010). The number of CCR genes differs significantly from that of species such as rice (26) (Kawasaki et al., 2006), pear (31) (Su et al., 2019), wheat (2) (Ma, 2007), tomato (2) (Rest et al., 2006), and corn (2) (Pichon et al., 1998). The quantitative differences among these different species also reflect that the *CCR* gene has undergone events such as ecological adaptation and historical evolution, and genomic replication events have occurred during the evolutionary process. Over time, homologous gene differentiation caused by gene duplication can promote the generation of new characteristics or functions among gene family members (Zafar et al., 2022). Twelve pairs of fragment repeating genes were identified in the *StCCR* gene family of potatoes, which may be related to the fact that the Atlantic potato is a homotetraploid and the underlying evolutionary driving force. Phylogenetic analysis revealed that the *StCCRs* subfamily 1 gene of potatoes has more special motifs and key structures consistent with *Arabidopsis thaliana*, and is more closely related to the evolution of CCR genes in most other angiosperms. This suggests that the *StCCRs* subfamily 1 gene of potatoes may be related to the true function of CCR proteins. Researchers believe that after a long period of evolution, not all members of the *CCR* family have been involved in the synthesis of lignin (Cheng et al., 2017). We speculate that the *SCCRs* subfamily 2 gene, which is not grouped with *AtCCRs*, may have lost some of its original functions and developed other new functions during the long process of evolution. What is rather interesting is that the genes of the above-mentioned subgroups are fragment repetitive genes and pairs of genes to each other.

Compared with *Arabidopsis thaliana*, potato *StCCRs* have more collinear gene pairs than the other three solanaceae plants, which indicates that the *CCR* gene family has a higher degree of conservation in solanaceae plants. These genomic similarity and difference patterns further reveal the species relationships and genomic evolutionary characteristics among solanaceae plants. Meanwhile, combined with the low Ka/Ks ratio in the stress analysis of the *StCCR* gene, it was found that the *StCCR* gene family was subjected to strong selection pressure and underwent strong purification selection, ultimately retaining favorable genetic variations. These results suggest that *StCCRs* are relatively conserved and functionally important genes. In the gene structure, the *StCCRs* subfamily has similar structures and the number of exons and introns between and within groups. This makes the more compact gene structure among them express more rapidly in response to external environmental stimuli, and the fewer the number of introns, the faster the expression speed of the gene when the environment changes, thereby enabling it to exert more effective functions (Kavas et al., 2016; Vadde, 2021).

Promoter cis-elements are important means for exploring gene functions and play a significant role in plant growth regulation. For example, the ethylene response factor AP2/ERF regulates *CCR* expression by responding to methyl jasmonate, SA and ABA reactions to mediate lignin synthesis (Ma et al., 2017). The promoter region of the *StCCR* gene in this study contains multiple cis-regulatory elements involved in plant hormones, light responses and environmental stress responses. Unlike ABA and IAA, the expression level increase of *StCCR* subfamily 1 gene was relatively low after GA3 treatment, while subfamily 2 gene had a continuous and significant response throughout the treatment process. This might be due to the fact that GA3 plays a prominent role in promoting plant growth, but its mechanism of action for improving plant stress resistance is not as significant as that of ABA and IAA. In a study on chili peppers, it was found that the expression level of the promoter of the *CCR* gene increased under ABA and GA3 treatments, while the expression level changed less under SA and MeJA treatments (Wu, 2022). The expression level of the *StCCRs* gene (except for the *StCCR6* gene) generally decreased with the extension of time under low-temperature treatment. The possible reason for the analysis might be to remove as much water as possible to avoid affecting the normal growth and development of plants due to factors such as low-temperature ice crystals. A similar situation also exists in *Arabidopsis thaliana* (Ji et al., 2015). On the contrary, when plants resist the effects of high temperature and drought, the *StCCR* gene increases the rate of lignin synthesis in plants by accelerating transcription and translation, and speeds up the degree of lignification, thereby reducing their own transpiration and water loss and lowering the damage caused by high temperature. During this process, the genes of *StCCR* subfamily 1 all respond. In poplar trees (De Meester et al., 2020), 12 *CCR* genes induced by high temperature are involved in the large-scale synthesis of lignin. Unlike temperature, most *StCCRs* have significant responses to salt and drought stress at 24 hours. Combined with the abiotic stress elements ABRE and MBS analyzed from the promoter elements of *StCCR2*, *StCCR4*, and *StCCR6* genes, this indicates that some *StCCRs* may play an important role when plants are subjected to salt and drought stress. In other species, both the *CCR* gene and the transcription factor MYB in the root system of melon seedlings were significantly expressed in the early response to salt stress (Wei et al., 2013). The expression level of the *CCR11* gene in Populus euphratica was also upregulated under conditions of salt and water deficiency (Hori et al., 2020). Under drought and salt stress conditions, the expression level of *HcCCR2* in jute plants reaches the highest point, and *HcCCR2* is expressed in roots, stems, leaves and flowers (Ghosh et al., 2014). The *CCR* gene shows differences in tissue expression among different plants (Wang, 2019; Guillermo et al., 1999; Mcinnes et al., 2002). In *Arabidopsis thaliana*, the expression level of the *AtCCR1* gene is the highest in the stem and relatively low in the leaves. However, *AtCCR2* does not participate in the constitutive expression of lignin, but is involved in the synthesis of chemical substances such as phenols, and participates in promoting the disease and stress resistance of plants after the formation of lignin (Lauvergeat et al., 2001). The expression levels of the *StCCR* gene in roots, stems and leaves show significant differences. The expression levels in stems and leaves are higher than those in roots. This result is consistent with the previous study that showed *TaCCR1* is not expressed in the roots but is mainly expressed in the stems and leaves (Lin et al., 2001), indicating that *StCCR* is involved in the growth and development process of potatoes. The previous differentiation and precipitation of elements related to cell differentiation in their promoters further emphasized their crucial role in plant development.

The subcellular localization results of the *StCCR6* gene indicated that the *StCCR6* protein was located in the cytoplasm, and this result was the same as that of the *CCR* gene localization results of species such as *Arabidopsis thaliana* (Ni et al., 2019; Liu et al., 2011; Miao et al., 2015). This is also consistent with the result that the *CCR* gene catalyzed the biosynthesis of lignin monomers in the cytoplasm and polymerized to form lignin macromolecules on the cell wall (Li et al., 2010), providing assistance for further explaining the function of the StCCR protein. Lignin can provide mechanical support for plants by increasing the hardness of the cell wall and enhancing the compressive strength of the cells, and it can also serve as a physical defense in the response against pathogen infection. In plants, technologies based on RNAi and VIGS have successfully silenced specific gene members without affecting the transcription of other closely related family members, or simultaneously silenced a few gene family members to overcome functional redundancy. In this study, the analysis results of lignin content and the expression levels of phenylpropane pathway genes related to lignin biosynthesis in WT plants and silenced plants all suggested that the *StCCR6* gene might affect the synthesis of lignin in plants, and the silencing of *StCCR6* might also affect the transcriptional levels of lignin synthesis pathway genes, thereby influencing the accumulation of lignin. This further affects the structure and function of the plant cell wall. Under biological stress, the situation of silencing is the same as that of wild plants. The contents of SOD, POD, and CAT enzymes increase to varying degrees after pathogen infection, indicating that the enzyme activity response of plants after inoculation is more sensitive, and by increasing the enzyme activity and content, the reactive oxygen species and MDA and other substances produced by plant stress are balanced and reduced. The difference between the two is that the increase in antioxidant enzyme content of silent plants is lower than that of wild plants, and their antioxidant capacity is somewhat weaker. And because the content of reactive oxygen species and other substances accumulated by the silent plants is relatively high, it may cause more serious damage to the plants.

In conclusion, the above analysis makes us believe that the research on the *CCR* gene family in potatoes has led to some new discoveries and understandings compared with other species in the past, and also provides a certain theoretical basis for the in-depth study of gene functions in the future. Although viral-induced gene silencing (VIGS) has been confirmed, no complementary experiments or functional validations (such as overexpression) have been conducted yet. In the future, we need more experimental results to strengthen this conclusion.

## 5 Conclusions

In this study, 10 members of the *StCCR* family of Atlantic potatoes were identified and analyzed. Their physicochemical properties were not significantly different, and they showed a high degree of conservation in terms of gene structure and evolutionary relationship. A total of 12 gene duplication events were found. Collinearity analysis showed higher homology with Solanaceae plants. Cis-element analysis revealed that the *StCCR* gene had a significant response to abiotic stress. Analysis of the tissue specificity of the *StCCR* gene and its expression pattern under abiotic stress using qRT-PCR indicated that the *StCCR* subfamily I (*StCCR2*, *StCCR4*, *StCCR6*, *StCCR7*) genes responded more significantly to various stresses. This implies that the potato cinnamoyl-Coenzyme A reductase protein has different functional differentiations, which is also helpful for screening valuable candidate *StCCRs* genes. After silencing *StCCR6*, it was found that the cell wall structure and lignin content of potatoes changed, and the leaves of the silenced plants suffered more severe oxidative damage after pathogen infection. These conclusions provide valuable information for further research on the biological role of the *StCCRs* gene in the stress growth process of potatoes and are of great significance to potato agricultural production.

## Data Availability

The datasets presented in this study can be found in online repositories. The names of the repository/repositories and accession number(s) can be found in the article/**Supplementary Material**.
